# Influence of Intra-Articular Tunnel Aperture Morphology on Clinical Outcomes and Graft Rerupture After ACL Reconstruction

**DOI:** 10.3390/jcm15010172

**Published:** 2025-12-25

**Authors:** Yusuf Iyetin, Emre Koraman, Mehmet Akan, Ismail Turkmen, Muhlik Akyurek

**Affiliations:** 1 Department of Orthopaedics and Traumatology, Pendik Bolge Hospital, 34890 Istanbul, Turkey; y.iyetin@gmail.com; 2 Department of Orthopaedics and Traumatology, Luleburgaz State Hospital, 39750 Kirklareli, Turkey; akanmehmet9@gmail.com; 3 Department of Orthopaedics and Traumatology, Klinikum Osnabruck, 49076 Osnabruck, Germany; dr.ismailturkmen@gmail.com; 4 Department of Orthopaedics and Traumatology, Maria-Josef Hospital, 48268 Greven, Germany; makyuerek@web.de

**Keywords:** anterior cruciate ligament reconstruction, intra-articular tunnel aperture, tunnel geometry, graft rerupture, clinical outcomes, graft survival

## Abstract

**Background/Objectives:** Variations in drill orientation during femoral and tibial tunnel creation can alter intra-articular tunnel aperture morphology in anterior cruciate ligament (ACL) reconstruction. Enlarged or irregular apertures may affect graft–tunnel conformity and graft mechanics. This study aimed to assess the relationship between intra-articular femoral and tibial tunnel aperture areas, postoperative clinical outcomes, and graft rerupture. This study specifically focuses on MRI-based measurement of intra-articular tunnel aperture area, a morphological parameter that has not been routinely evaluated in previous ACL reconstruction studies and differs conceptually from tunnel diameter or drilling angles. **Methods:** This retrospective case–control study included patients who underwent primary ACL reconstruction with an 8 mm hamstring autograft using the anteromedial portal technique. All patients completed a minimum 2-year follow-up and postoperative MRI. Femoral and tibial intra-articular aperture areas were measured on MRI Clinical outcomes (Lysholm scores and KOOSs) in patients with intact grafts. Patients were categorized into intact-graft and rerupture groups. Correlation analyses were used to evaluate associations between aperture areas and clinical outcomes. Logistic regression identified predictors of rerupture. **Results:** A total of 152 patients met the inclusion criteria, including 13 with graft rerupture. In the intact-graft group, mean femoral and tibial aperture areas were 127.34 ± 8.92 mm^2^ and 138.33 ± 7.08 mm^2^, respectively. Both aperture areas demonstrated significant negative correlations with Lysholm scores and KOOSs. Patients with rerupture had significantly larger femoral (145.26 ± 4.22 mm^2^) and tibial (158.02 ± 2.88 mm^2^) aperture areas (*p* < 0.001 for both). Logistic regression identified tibial aperture area as a significant predictor of rerupture. **Conclusions:** Larger intra-articular tunnel aperture areas were associated with inferior functional outcomes, and increased tibial aperture area correlated significantly with graft rerupture. Aperture morphology may represent an important factor influencing graft integrity and postoperative recovery after ACL reconstruction.

## 1. Introduction

Although anterior cruciate ligament (ACL) reconstruction has been widely performed for decades, substantial controversy remains regarding optimal femoral and tibial tunnel creation techniques and the interaction between graft dimensions and tunnel aperture morphology [1]. Biomechanical studies have demonstrated that reproducing the native ACL anatomy improves postoperative knee kinematics and stability [2]. For this reason, accurate tunnel preparation is essential for reliable graft incorporation—especially when using soft-tissue grafts—and for maintaining normal postoperative knee biomechanics [3]. While tibial tunnel creation tends to follow more standardized principles, femoral tunnel placement continues to be a major source of variation and debate [4]. Prior research has shown that even small deviations in femoral tunnel orientation, particularly in the sagittal plane, can negatively affect clinical outcomes [5].

Experimental studies comparing anteromedial (AM) portal and transtibial drilling techniques further indicate that femoral tunnel length and geometry vary substantially depending on drilling angles in the coronal and sagittal planes [6]. Although tunnel diameter and drilling angles have been extensively investigated in the literature, the clinical significance of intra-articular tunnel aperture morphology—representing the graft-tunnel interface at the joint level—remains poorly understood.

The aim of this study was to evaluate how enlargement of intra-articular femoral and tibial tunnel apertures affects clinical outcomes and graft rerupture rates within a standardized cohort. It was hypothesized that variations in drill inclination in the coronal and sagittal planes may generate more elliptical and enlarged tunnel apertures, and increased intra-articular tunnel aperture area, reflecting deviations in drill orientation during tunnel creation, would be associated with inferior functional outcomes and a higher risk of graft rerupture.

## 2. Materials and Methods

Ethical approval was obtained from the Institutional Internal Review Board and Clinical Research Ethical Committee (Date: 25 October 2023, No: 2023/0722). Written informed consent was obtained from all participants. All patients presenting with an acute ACL injury between January 2018 and January 2022 were retrospectively reviewed.

### 2.1. Patient Selection

Patients aged > 18 years who underwent primary ACL reconstruction using a button fixation system (Endobutton CL Ultra Fixation device with a 9 × 30 mm Biosure Regenesorb interference screw, Smith & Nephew, Andover, MA, USA; ACL TightRope II Implant System with a 9 × 30 mm biocomposite interference screw, Arthrex, Naples, FL, USA) were screened. Only patients who received a hamstring autograft with an 8 mm graft diameter were included to ensure uniformity in drill diameter and to minimize variation in tunnel aperture morphology. Patients were required to have completed two years of follow-up, including a 2-year postoperative magnetic resonance imaging (MRI).

Exclusion criteria were defined to maintain a homogeneous cohort and reduce confounding. Patients were excluded if they had:

- A history of previous fractures;
- Body mass index (BMI) ≥ 30;
- Varus/valgus malalignment;
- Associated meniscal or ligamentous injuries or osteochondral lesions;
- Advanced osteoarthritis (Kellgren–Lawrence grade ≥ 2);
- Abnormal patellar tracking or abnormal tibial slope;
- Intraoperative requirement for notchplasty;
- Postoperative infection;
- Loss to follow-up;
- Non-compliance with rehabilitation;
- Major trauma or contact injury after ACL reconstruction, as such mechanisms could obscure whether rerupture was attributable to tunnel-related factors or new injury events;
- Quadriceps atrophy on thigh-circumference assessment.

A retrospective design was chosen to allow analysis of a relatively large and standardized cohort with uniform graft size and long-term postoperative MRI follow-up.

### 2.2. Surgical Technique

All procedures were performed by the same two senior surgeons. Following routine arthroscopic inspection through anterolateral and anteromedial portals, hamstring tendons were harvested and prepared. With the knee in 120° hyperflexion, the femoral tunnel was created at the anatomical footprint through the AM portal. Subsequently, with the knee flexed at 90°, the tibial tunnel was drilled using a guide set at 55° in the sagittal plane, targeting the ACL footprint posterior to the anterior horn of the lateral meniscus. The prepared graft was passed through the tunnels using a suspensory button, and tibial fixation was achieved with a bioscrew. Femoral coronal/sagittal drill inclination and tibial coronal drill inclination were not measured intraoperatively, as the aim of this study was to evaluate the clinical consequences of the final intra-articular aperture morphology rather than to quantify specific drilling angles. Variability in tunnel orientation is known to occur during AM portal drilling in routine practice, and aperture morphology was therefore used as a clinically relevant indirect marker of these deviations.

### 2.3. Patient Assessment

Preoperative and postoperative weight-bearing anteroposterior and lateral radiographs at 30° knee flexion were obtained. All MRI examinations were performed at the second postoperative year, allowing assessment of final intra-articular tunnel aperture morphology rather than early postoperative changes. Intra-articular aperture morphology was intentionally selected as an integrated surrogate reflecting drill orientation, drill wobble, and cortical engagement, thereby capturing real-world surgical variability rather than isolated drilling angles. MRI examinations were performed using a 1.5-T system (MR360 Optima, GE Healthcare, Chicago, IL, USA) with a standard extremity coil. T1-weighted sagittal, T2-weighted axial, and fat-suppressed proton density sagittal and coronal sequences were obtained (FOV 18.0 cm; spacing 1 mm; slice thickness 2 mm). These parameters provided adequate resolution for consistent aperture evaluation while avoiding unnecessary radiation exposure associated with computed tomography (CT), which is not routinely used in postoperative ACL follow-up.

Patients were categorized into two groups:

1. Rerupture group;
2. Intact-graft group.

Rerupture was defined as graft failure occurring without a history of major trauma or contact injury. Functional scores (Lysholm and Knee injury and Osteoarthritis Outcome Score (KOOS)) were evaluated only in the intact-graft group, as rerupture and subsequent instability or revision would introduce substantial bias into patient-reported outcomes.

Demographic variables, femoral and tibial aperture areas, and associations between aperture morphology, clinical scores, and rerupture were analyzed.

The tibial slope was measured on preoperative radiographs using a method previously described in the literature [7].

### 2.4. Aperture Measurement

**Figure 1:**
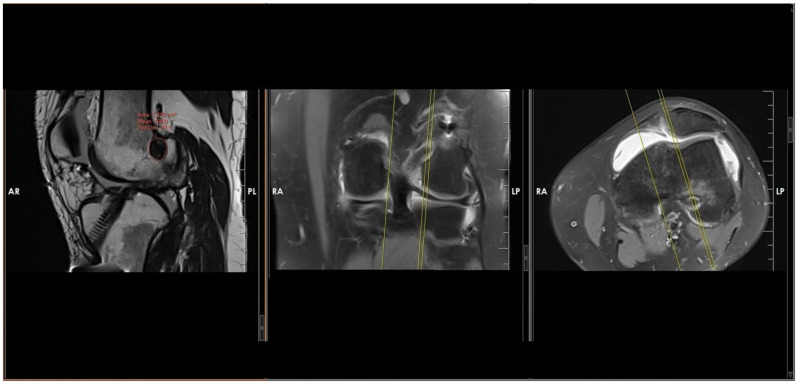
The femoral tunnel aperture on the joint surfaces of the femur was evaluated following anterior cruciate ligament reconstruction. Reference lines were used to identify the exact tunnel orientation, and the aperture area at the joint interface was measured using a freehand measurement tool (Uppercase letters and yellow lines are automatically generated by the MRI analysis software and represent measurement values and reference boundaries).

**Figure 2:**
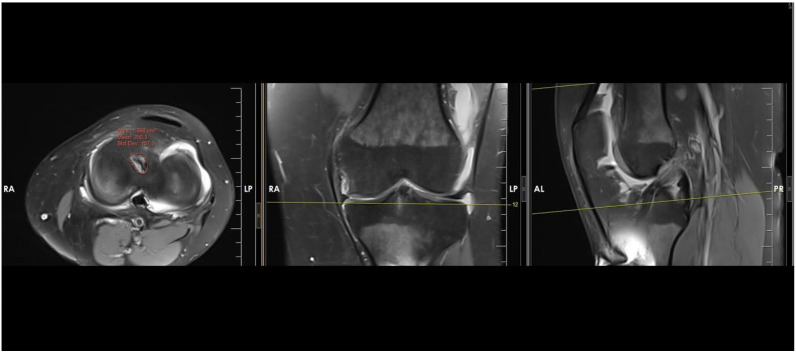
The tibial tunnel aperture was analyzed on the joint surfaces of the tibia post-anterior cruciate ligament reconstruction. The tunnel aperture area at the joint interface was precisely measured using reference lines and a freehand measurement tool for accuracy (Uppercase letters and yellow lines are automatically generated by the MRI analysis software and represent measurement values and reference boundaries).

Intra-articular femoral and tibial tunnel apertures were evaluated using MRI. Measurements were performed on OsiriX MD (Version 12.0, Pixmeo, Bernex, Switzerland) using the closed-polygon freehand tool, tracing the outer cortical margins of the bone tunnel to exclude soft-tissue structures. For each tunnel, the maximum aperture area was identified by reviewing the sagittal, coronal, and axial planes using predefined anatomical reference lines to ensure consistent alignment. Measurements were taken from the MRI slice demonstrating the largest cross-sectional osseous aperture at the joint interface (Figure 1 and Figure 2).

Two experienced observers independently performed all measurements, each repeating them three times. Observers were blinded to each other’s measurements and to patient identity. Inter- and intra-observer reliability were assessed using intraclass correlation coefficients (ICCs), with the study demonstrating excellent agreement (ICC = 0.91). This high reproducibility minimized potential variability associated with manual tracing.

### 2.5. Statistical Analysis

NCSS software (Number Cruncher Statistical System version 07, 2007, Kaysville, UT, USA) was used for all analyses. Frequencies, percentages, means, and standard deviations were calculated for demographic data and radiological and clinical outcomes. The Kolmogorov-Smirnov test was used to determine normality. Comparisons of these data were performed using independent samples *t* tests and chi-squared tests in patients with or without rerupture. The Spearman correlation test was used to determine the relationship between femoral and tibial tunnel aperture areas and clinical outcomes in patients without rerupture. The relationships between rerupture and femoral and/or tibial aperture areas were analyzed by logistic regression analysis, and *p* < 0.05 was considered to indicate statistical significance. The sample size was calculated using G*Power 3. Based on the calculations, a minimum sample size of 75 patients was required to observe a correlation between the Lysholm score and the femoral aperture and tibial aperture [type 1 error (α) of 0.05, power (1 − β) of 0.80] [8].

## 3. Results

**Figure 3:**
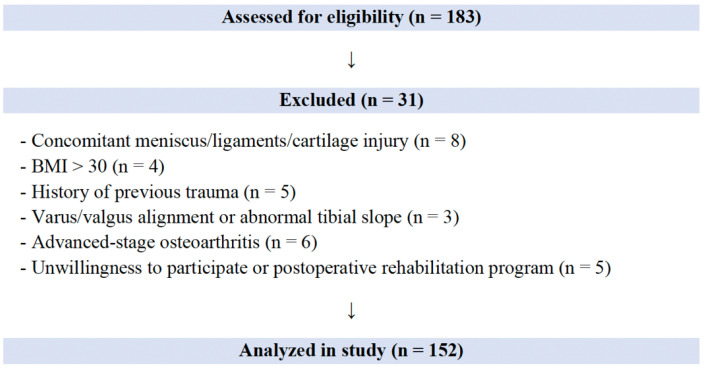
The flowchart of patients included in and excluded from the study.

A total of 152 patients met the inclusion criteria and were included in the study, of whom 13 (8.5%) experienced graft rerupture requiring revision surgery (Figure 3).

The demographic characteristics of patients with intact grafts and those with rerupture were comparable, with no significant differences in age, sex distribution, laterality, or BMI (Table 1).

### 3.1. Radiological Measurements and Clinical Outcomes

Among patients with intact grafts, the mean intra-articular femoral tunnel aperture area was 127.34 ± 8.92 mm^2^, and the tibial tunnel aperture area was 138.33 ± 7.08 mm^2^. The mean Lysholm score was 85.17 ± 11.78, and the KOOS was 85.62 ± 11.49 at the 2-year follow-up.

In patients with rerupture, the mean femoral aperture area was 145.26 ± 4.22 mm^2^, and the tibial aperture area was 158.02 ± 2.88 mm^2^. Both femoral and tibial aperture areas were significantly larger in the rerupture group than in the intact-graft group (*p* < 0.001 for both comparisons) (Table 2).

### 3.2. Correlations Between Aperture Morphology and Clinical Scores

In the intact-graft group, Spearman correlation analysis demonstrated a strong negative correlation between the femoral aperture area and both Lysholm scores (r = −0.938, *p* < 0.001) and KOOSs (r = −0.925, *p* < 0.001). Similarly, tibial aperture area showed a moderate negative correlation with Lysholm scores (r = −0.607, *p* < 0.001) and KOOSs (r = −0.613, *p* < 0.001) (Table 3).

Logistic regression revealed that tibial aperture area was significantly associated with rerupture (OR = 1.8485; 95% CI: 1.1537–2.9617; *p* = 0.0106). Femoral aperture area did not reach statistical significance (*p* = 0.1779) (Table 4).

## 4. Discussion

The principal finding of this study was that larger intra-articular tunnel aperture areas in both the femur and tibia were associated with inferior clinical outcomes, and that an increased tibial aperture area demonstrated a significant relationship with graft rerupture. These results support the hypothesis that variations in drill inclination during tunnel creation—which can produce more elliptical and enlarged intra-articular apertures—may compromise graft–tunnel conformity and contribute to graft micromotion within the joint space. This mechanical mismatch appears to have meaningful clinical implications. Although biological tunnel widening is a well-recognized phenomenon following ACL reconstruction, it typically occurs during the early postoperative period and reflects progressive, circumferential changes along the tunnel length. In contrast, the present study focused specifically on intra-articular tunnel aperture morphology at the joint interface evaluated two years after surgery. This localized geometric alteration, characterized by increased aperture area and ellipticity, is more likely influenced by mechanical factors such as drill orientation relative to the bone cortex rather than biological widening alone.

Femoral tunnel orientation has long been a point of debate in ACL reconstruction, particularly when comparing transtibial and independent techniques such as the AM portal approach [9]. Prior studies have emphasized the biomechanical advantages of anatomically positioning the femoral tunnel to reproduce native ACL orientation and improve postoperative kinematics [10,11]. However, different drilling strategies may alter the morphology of the intra-articular aperture, thereby affecting the stresses transmitted to the graft as it bends over the cortical edge. Excessive bending stress at the aperture has been suggested as a potential contributor to graft attrition over time [12].

Most previous investigations have focused on femoral graft bending angles, with less emphasis on the tibial aperture [13]. Studies comparing the transtibial and AM techniques have shown that the tibial aperture created with the transtibial approach tends to be larger and more elliptical, whereas the AM technique yields a more circular aperture [14,15]. Although biomechanical comparisons between these techniques have demonstrated no significant differences in stiffness, contact area or pressure, peak contact pressures were found to be higher with the AM technique [9]. These findings suggest that even subtle alterations in aperture geometry may influence the mechanical environment surrounding the graft.

On the tibial side, fixation characteristics may further influence aperture-related graft mechanics. Suspensory fixation is commonly used on the femoral side, whereas tibial fixation relies on an interference screw positioned within the tunnel [16]. Due to its conical design, the screw may not fully occupy widened or irregularly shaped tunnel apertures, potentially allowing increased graft–tunnel micromotion, particularly near the joint line. Techniques that attempt to position the screw closer to the aperture may mitigate this effect on the tibial side, but no similar adjustment is possible for the femur [17]. An important finding of the present study was that, although both femoral and tibial aperture areas correlated with functional outcomes, only tibial aperture area independently predicted graft rerupture. This observation may be explained by biomechanical differences between the two tunnel sites. The tibial tunnel is exposed to greater graft motion, increased bending and shear stresses, and a longer graft–tunnel interface close to the joint line during knee motion. These biomechanical conditions may amplify the adverse effects of an enlarged or elliptical tibial aperture, potentially increasing graft strain and susceptibility to failure.

Additional biomechanical factors may contribute to the formation of irregular or enlarged apertures. Drill wobble, guide wire deviation, and variations in drill angle relative to the cortical surface have been shown to affect tunnel dimensions and lead to aperture inconsistencies [18,19,20]. Prior work has also established the importance of graft–tunnel conformity for tendon-to-bone healing. Experimental models have demonstrated that minimal gaps between the graft and tunnel facilitate collagen fiber integration, whereas mismatches greater than approximately 1.8 mm reduce interface strength and lead to disorganized fibrovascular tissue [21]. These findings are consistent with the present results, indicating that even modest increases in intra-articular aperture size may interfere with early graft healing and adversely influence long-term clinical outcomes. In the present study, this relationship was reflected by particularly strong correlations between aperture area and functional outcomes, which may be partly explained by the methodological homogeneity of the cohort and the use of a standardized graft diameter, thereby minimizing confounding variability.

Radiological studies evaluating postoperative tunnel diameter have historically reported inconsistent correlations with clinical results, likely due to limitations in detecting subtle changes using standard imaging modalities [22,23]. The present study differs by focusing specifically on the intra-articular aperture—the region of greatest biomechanical relevance—rather than the entire tunnel. Our results suggest that aperture morphology, rather than tunnel diameter alone, may be more predictive of graft behavior within the joint and may better explain the mechanisms underlying graft failure and suboptimal functional recovery. These findings underscore the importance of meticulous drill positioning during both femoral and tibial tunnel preparation to minimize unintended aperture enlargement or ellipticity.

Overall, the data indicate that intra-articular aperture morphology is an under-recognized factor influencing graft biomechanics and clinical outcomes following ACL reconstruction. From a clinical perspective, these findings suggest that surgeons should be aware that even subtle deviations in drill orientation may result in clinically relevant enlargement or ellipticity of the intra-articular aperture. Although the present study demonstrates an association rather than causation, careful attention to drill positioning during tunnel preparation may represent a potentially modifiable technical factor with implications for graft integrity and postoperative function. Future work should include three-dimensional analyses of aperture geometry and prospective evaluation of how specific drilling angles affect graft stress distribution and healing dynamics.

This study has several limitations. First, its retrospective and non-randomized design introduces an inherent risk of missing data and selection bias. Although strict inclusion criteria were applied to create a homogeneous cohort, residual confounding cannot be entirely excluded. Also, the relatively small number of rerupture events should be considered when interpreting regression analyses, and results should be viewed with appropriate caution. Second, intraoperative drill inclination in the coronal and sagittal planes was not directly measured. As a result, the relationship between drilling orientation and intra-articular aperture morphology was inferred indirectly rather than being quantitatively established. Additionally, aperture area measurements were based on two-dimensional MRI slices and may not fully capture the three-dimensional complexity of tunnel geometry. Third, although CT would provide more precise osseous measurements, it was not used due to radiation concerns, leading to reliance on MRI-based assessments. The imaging protocol, however, was standardized across all patients and allowed consistent aperture analysis. In addition, the requirement for a 2-year postoperative MRI may have introduced selection bias, as patients with persistent symptoms could be more likely to undergo follow-up imaging; nevertheless, identical inclusion criteria were uniformly applied, preserving the internal validity of the comparative analyses. Fourth, the study cohort was limited to patients with an 8 mm graft and drill diameter to ensure methodological uniformity. While this approach strengthens internal validity, it restricts the generalizability of the findings to other graft sizes and drilling conditions. Larger graft diameters may produce different tunnel geometries and could demonstrate different susceptibility to aperture-related mismatch. Finally, patients with associated meniscal pathology were excluded to minimize confounding effects on clinical outcomes. Although this improved cohort homogeneity, it may limit the applicability of the results to the broader ACL reconstruction population. Moreover, although patients with major trauma or contact injuries after ACL reconstruction were excluded, the exact biomechanical mechanisms leading to graft rerupture cannot be fully standardized across individuals. Future prospective studies incorporating intraoperative navigation, 3D imaging, or real-time angle tracking may help more precisely define the relationships between drill orientation, aperture geometry, and clinical outcomes.

## 5. Conclusions

This study found that larger intra-articular femoral and tibial tunnel aperture areas were accompanied by less favorable clinical outcomes following ACL reconstruction, and that increased tibial aperture area was observed more frequently in patients with graft rerupture. These observations highlight the clinical relevance of intra-articular aperture morphology and suggest that unintended enlargement or geometric alteration of the tunnel aperture—particularly on the tibial side—may be an important technical consideration during tunnel preparation. Careful attention to aperture morphology may therefore have implications for graft integrity and postoperative functional recovery.

## Figures and Tables

**Table 1:** Patient demographic variables.

	Patients with Intact Grafts	Patients with Rerupture	*p*
Age, years mean ± SD (Min–Max)	32.35 ± 10.03 (18–55)	32.85 ± 11.32 (18–52)	0.44 ^ϕ^
Male/female sex (% Percentage)	51/27 (65.39–34.61%)	7/6 (53.84–46.16%)	0.62 ^λ^
Right/left side (% Percentage)	38/40 (48.72–51.28%)	5/8 (38.46–61.54%)	0.69 ^λ^
BMI mean ± SD (Min–max)	21.21 ± 1.81 (18–24.5)	21.46 ± 2.18 (18–24.5)	0.32 ^ϕ^

^ϕ^ Independent samples *t* test, ^λ^ Chi-squared test, SD: Standard deviation, Min: Minimum, Max: Maximum.

**Table 2:** Radiological measurements of apertures and functional outcomes of patients with intact grafts and reruptures.

	Patients with Intact Grafts	Patients with Rerupture	*p*
Variables	Mean ± SD (Min–Max)	Mean ± SD (Min–Max)	
Femoral aperture area	127.34 ± 8.92 (113.27–155.68)	145.26 ± 4.22 (138.49–151.71)	<0.001 *^ϕ^
Tibial aperture area	138.33 ± 7.08 (129.31–162.86)	158.02 ± 2.88 (154.12–163.14)	<0.001 *^ϕ^
Lysholm score	85.17 ± 11.78 (30–100)		
KOOS	85.62 ± 11.49 (32.1–100)		

* *p* < 0.05, ^ϕ^ Independent samples *t* test, SD: Standard deviation, Min: Minimum, Max: Maximum, KOOS: Knee Injury and Osteoarthritis Outcome Score.

**Table 3:** Correlations between radiological and functional outcomes (patients with intact grafts).

Spearman’s Rho		Femoral Aperture Area	Tibial Aperture Area
**Lysholm score**	r	−0.938	−0.607
	*p*	<0.001 *	<0.001 *
**KOOS**	r	−0.925	−0.613
	*p*	<0.001 *	<0.001 *

* *p* < 0.05, KOOS: Knee Injury and Osteoarthritis Outcome Score.

**Table 4:** Relationships between femoral and tibial aperture areas and rerupture.

Variable	Coefficient	Standard Error	*p* Value	Odd’s Ratio	95% Confidence Interval
Femoral aperture area	−0.2483	0.1843	0.1779	0.7801	(0.5436–1.1196)
Tibial aperture area	0.6144	0.2405	0.0106 *	1.8485	(1.1537–2.9617)
Constant	−59.7217	18.3721	0.0012		

* *p* < 0.05.

## Data Availability

The raw data supporting the conclusions of this article will be made available by the authors on request.

## Abbreviations

The following abbreviations are used in this manuscript:

ACL	Anterior cruciate ligament
AM	Antermedial
MRI	Magnetic resonance imaging
BMI	Body mass index
CT	Computed tomography
KOOS	Knee injury and Osteoarthritis Outcome Score
ICC	Intraclass correlation coefficients
NCSS	Number Cruncher Statistical System
